# Supplementary material for: Germline Single-Nucleotide Polymorphism GFI1-36N Causes Alterations in Mitochondrial Metabolism and Leads to Increased ROS-Mediated DNA Damage in a Murine Model of Human Acute Myeloid Leukemia
Source: Biomedicines. 2025 Jan 5;13(1):107. doi: 10.3390/biomedicines13010107 (PMC11762481; doi:10.3390/biomedicines13010107)
Supplement: Supplementary file 1 [file biomedicines-13-00107-s001.zip › biomedicines-3341422-supplementary.pdf]

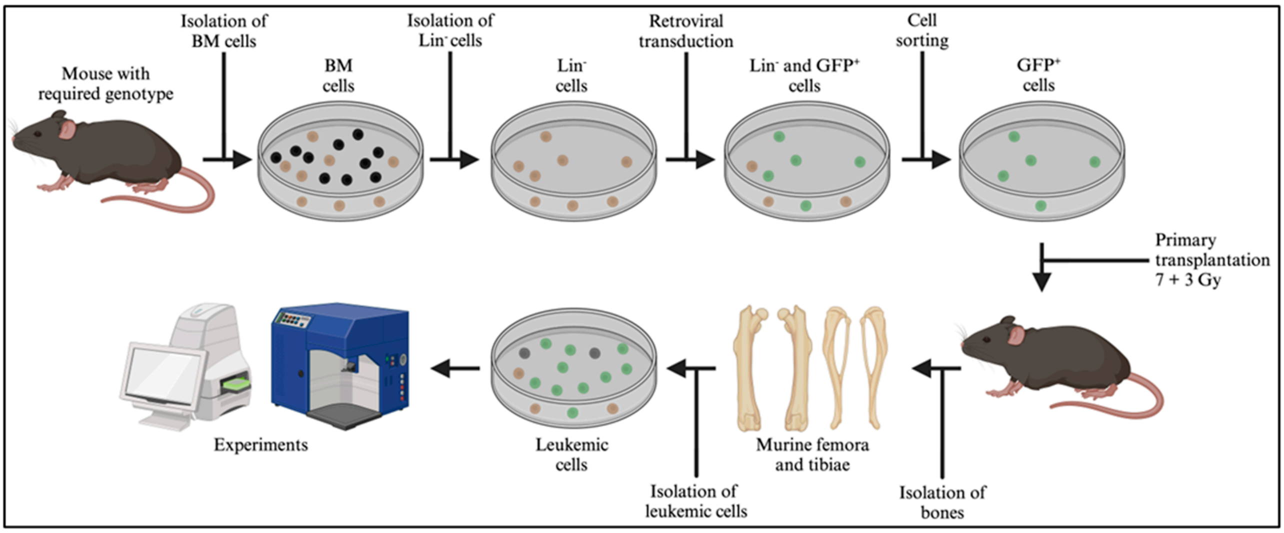

Figure S1. Generation of *MLL-AF9* leukemic *GFI1-36S* and *GFI1-36N* mice by primary bone marrow (BM) transplantation. During primary BM transplantation, Lin^- cells were isolated from *GFI1-36S* or *GFI1-36N* mice and transduced using an *MCSV-MLL-AF9-IRES-GFP* plasmid. The Lin^- cells were selected and were then injected into lethally irradiated (7 + 3 Gy) mice. Following the manifestation of leukemia symptoms, the mice were euthanized, the bones of the lower extremity were removed, and the malignant BM cells were flushed out. The leukemic cells were used either for experiments or for secondary BM transplantation.

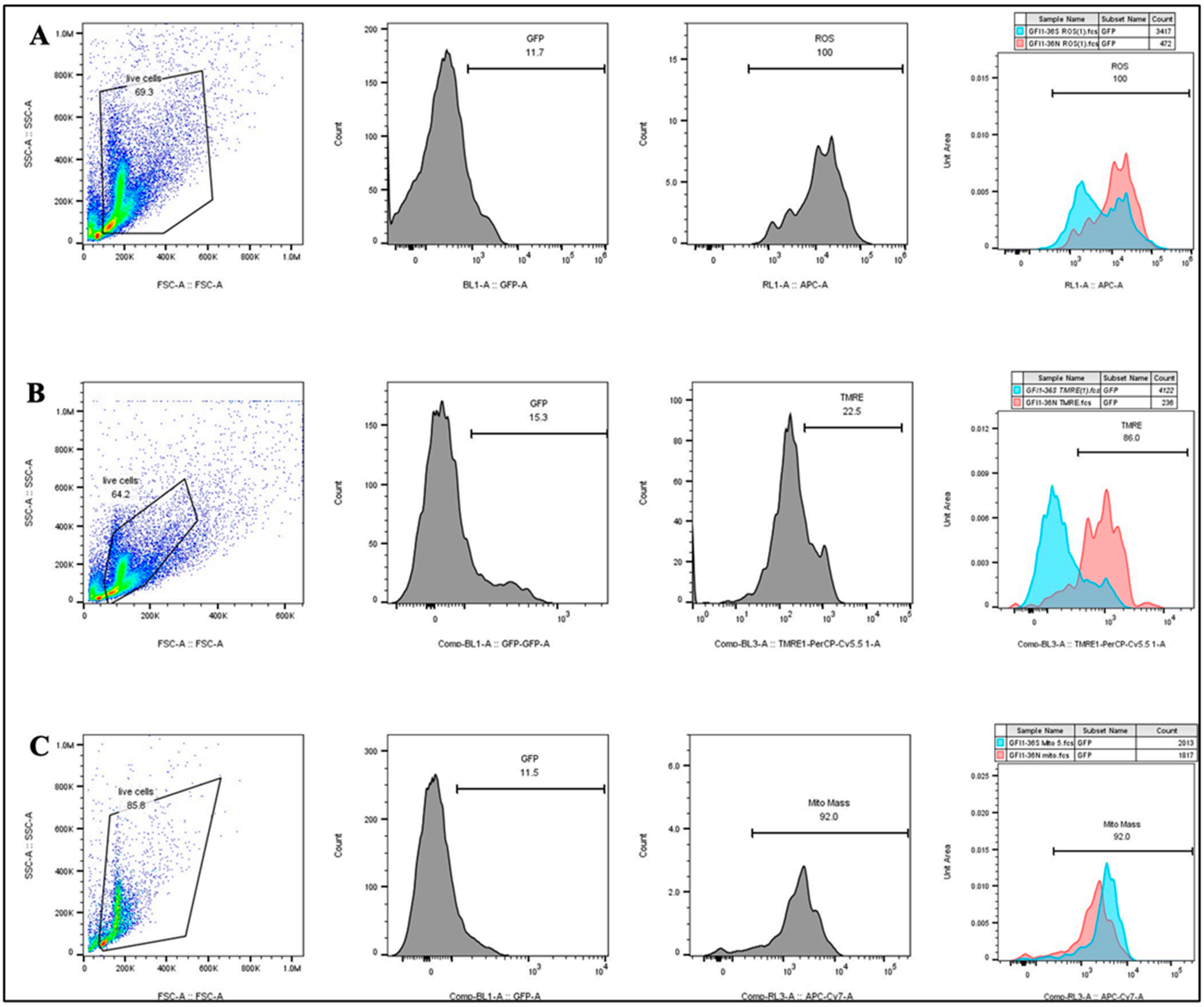

Figure S2. Flow cytometry discovers alterations in mitochondrial metabolism. Flow cytometry analysis of **A:** reactive oxygen species (ROS), **B:** mitochondrial membrane potential (MMP) using TMRE staining, and **C:** mitochondrial mass (mito-mass). Left: gating strategy for living cells. Middle-left: histogram showing GFP⁺ cells. Middle-right: histogram showing ROS, MMR, and mito-mass gating. Right: histogram overlay showing difference between *GFI1-36N* cells (red) compared to *GFI1-36S* cells (blue). $n = 1$.

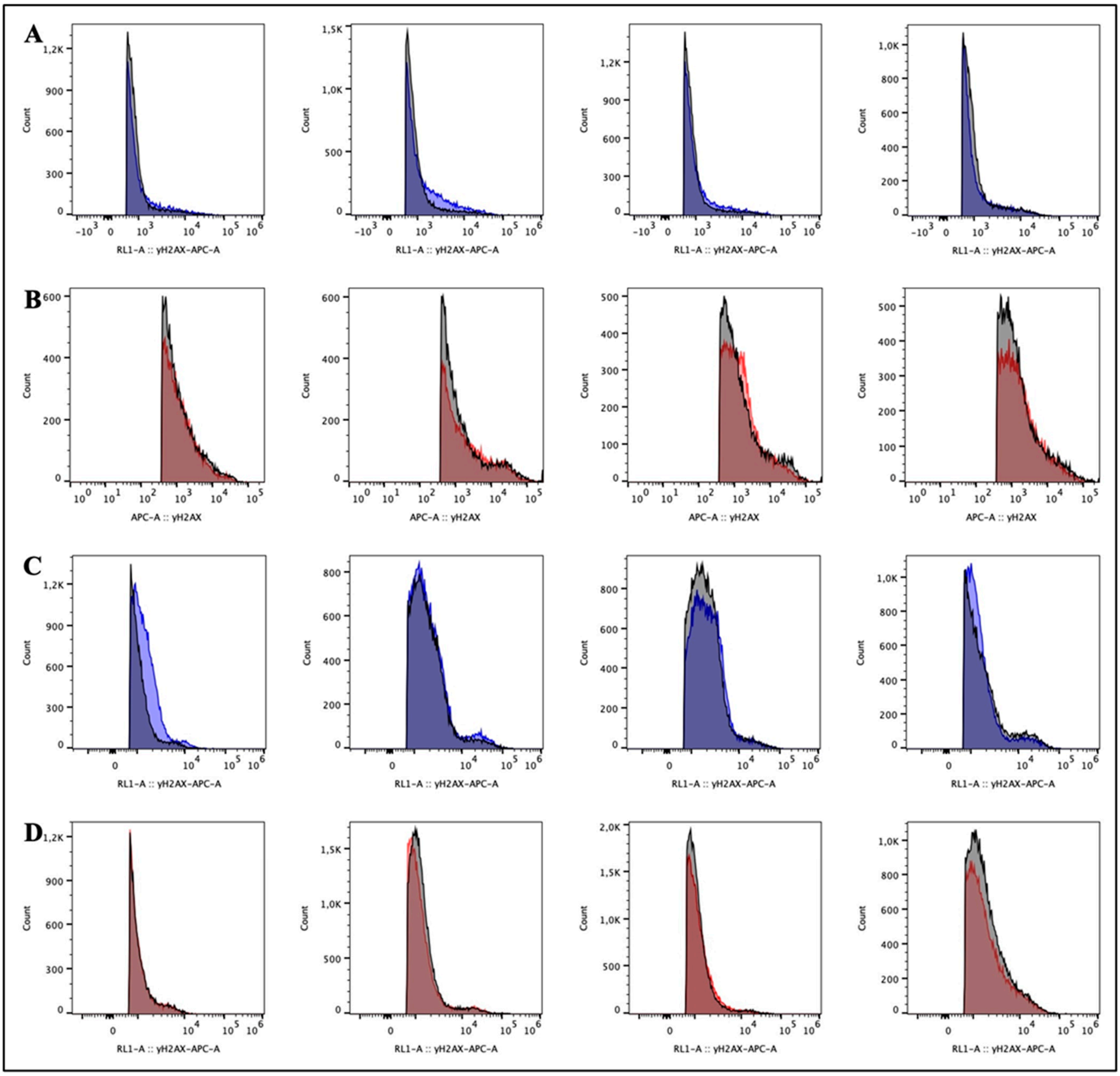

Figure S3. γ-H2AX⁺ *GFI1-36S* and *GFI1-36N* cells after treatment with N-acetylcysteine (NAC) or α-tocopherol (αT). A: Untreated (black) and NAC-treated (blue) *GFI1-36S* leukemic cells without irradiation (left), respectively 30 min (center-left), 60 min (center-right) and 120 min (right) after irradiation with 3 Gy. **B:** Untreated (black) and NAC-treated (red) *GFI1-36N* leukemic cells without irradiation (left), respectively 30 min (center-left), 60 min (center-right) and 120 min (right) after irradiation with 3 Gy. **C:** Untreated (black) and αT-treated (blue) *GFI1-36S* leukemic cells without irradiation (left), respectively 30 min (center-left), 60 min (center-right) and 120 min (right) after irradiation with 3 Gy. **D:** Untreated (black) and αT-treated (red) *GFI1-36N* leukemic cells without irradiation (left), respectively 30 min (center-left), 60 min (center-right) and 120 min (right) after irradiation with 3 Gy. n = 1.

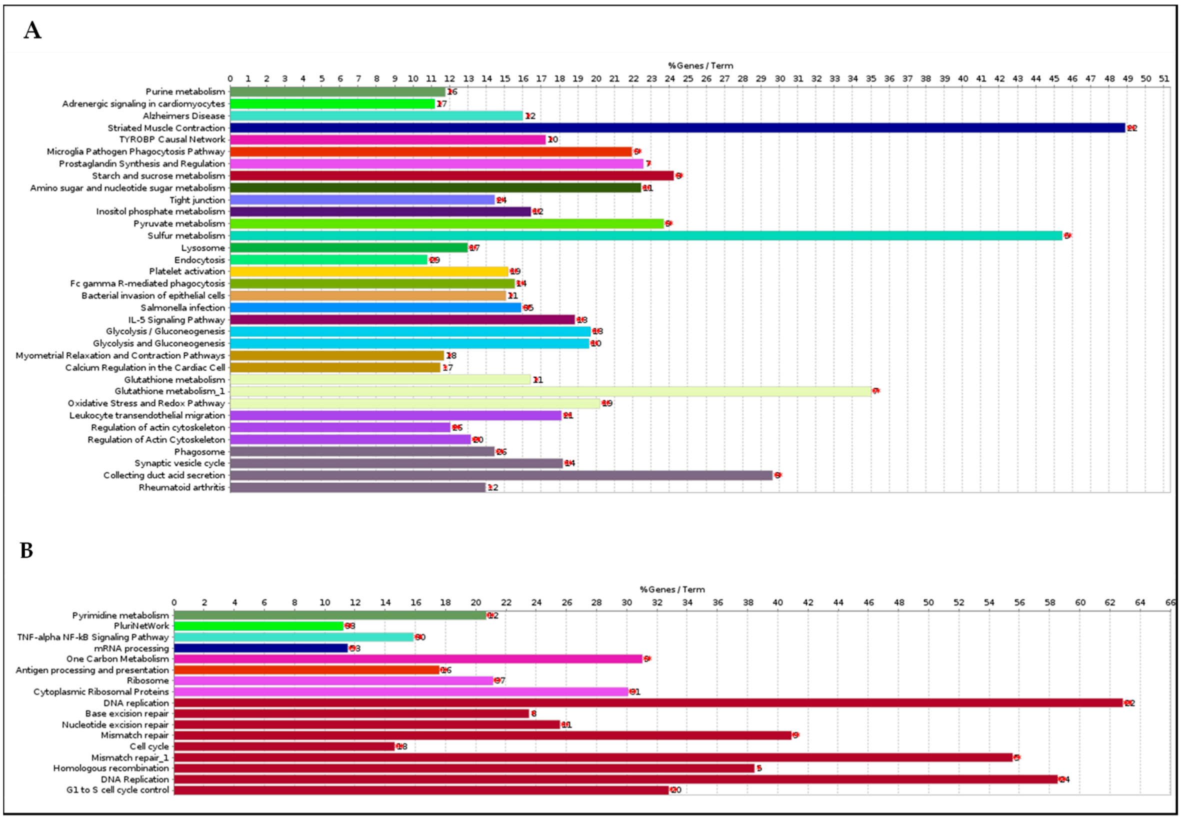

Figure S4. Altered pathways in *GFI1-36N-MLL-AF9* cells detected by proteomics. A: Down- and B: upregulated pathways in *GFI1-36N* leukemic cells; $n = 2 – 3$.

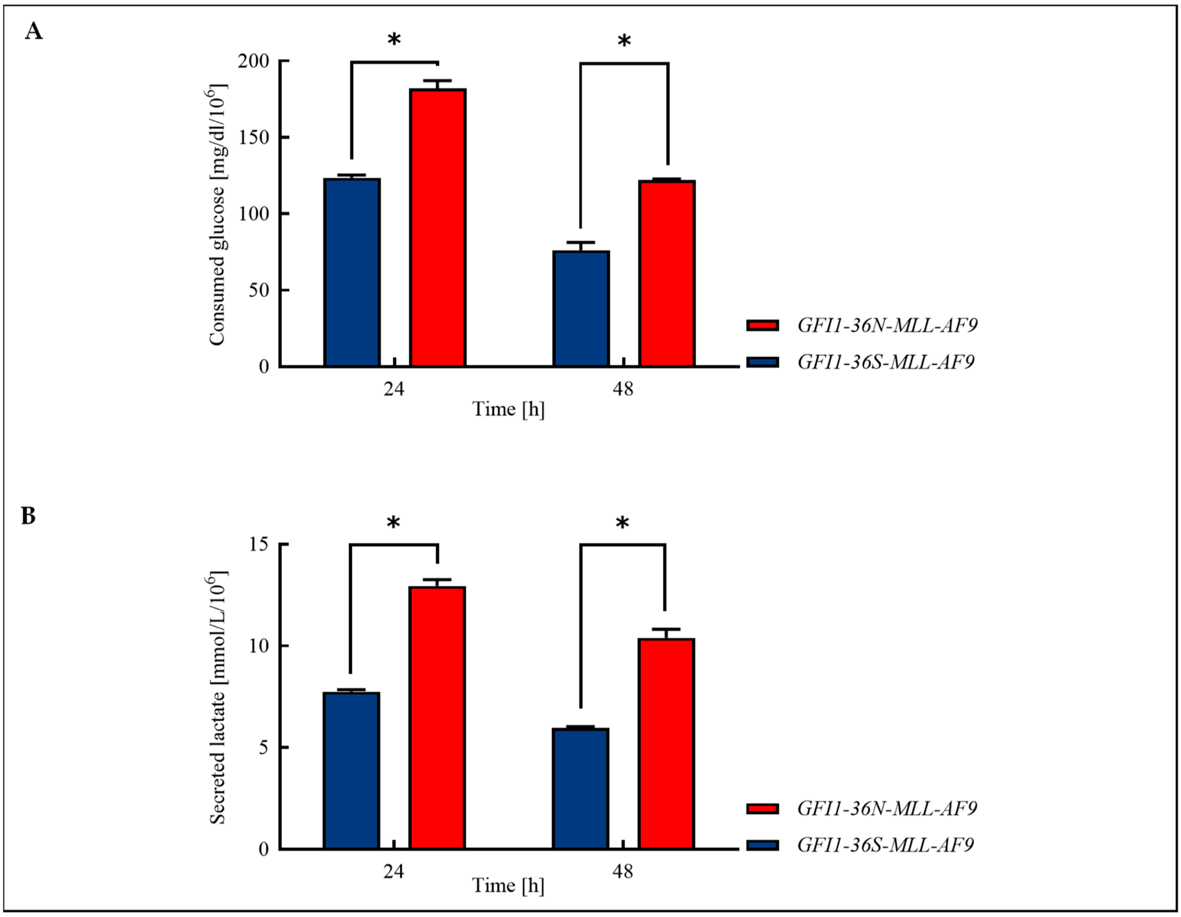

Figure S5. Glucose consumption and lactate secretion in *MLL-AF9* leukemic cells. Increased **A:** consumed glucose and **B:** secreted lactate levels in *GFI1-36N-MLL-AF9* cells. Mean ± SEM; p* ≤ 0.05; *n* = 2.
